# Silencing of SAA1 inhibits palmitate- or high-fat diet induced insulin resistance through suppression of the NF-κB pathway

**DOI:** 10.1186/s10020-019-0075-4

**Published:** 2019-05-06

**Authors:** Yong Wang, Feng Cao, Yang Wang, Gang Yu, Ben-Li Jia

**Affiliations:** Department of Gastrointestinal Surgery, the Second Hospital of Anhui Medical University, No. 678, Furong Road, Economic and Technological Development Zone, Hefei, 230601 Anhui Province People’s Republic of China

**Keywords:** Serum amyloid A-1, NF-κB pathway, Insulin resistance, Palmitate, High-fat diet

## Abstract

**Background:**

Obesity is one of the leading causes of insulin resistance. Accumulating reports have highlighted that serum amyloid A-1 (SAA1) is a potential candidate that is capable of attenuating insulin resistance. Hence, we conducted the current study with aims of investigating our proposed hypothesis that silencing SAA1 could inhibit the progression of obesity-induced insulin resistance through the NF-κB pathway.

**Methods:**

Gene expression microarray analysis was initially performed to screen differentially expressed genes (DEGs) associated with obesity. Palmitate (PA)-induced insulin resistance Huh7 cell models and high-fat diet (HFD)-induced mouse models were established to elucidate the effect of SAA1/Saa1 on insulin resistance. The NF-κB pathway-related expression was subsequently determined through the application of reverse transcription quantitative polymerase chain reaction (RT-qPCR) and Western blot analysis.

**Results:**

Saa1 was identified as an obesity-related gene based on the microarray data of GSE39549. Saa1 was determined to be highly expressed in HFD-induced insulin resistance mouse models. PA-induced Huh7 cells, treated with silenced SAA1 or NF-κB pathway inhibition using BAY 11–7082, displayed a marked decrease in both Saa1 and SOCS3 as well as an elevation in 2DG, IRS1 and the extent of IRS1 phosphorylation. HFD mice treated with silenced Saa1 or inhibited NF-κB pathway exhibited improved fasting blood glucose (FBG) levels as well as fasting plasma insulin (FPI) levels, glucose tolerance and systemic insulin sensitivity. Saa1/SAA1 was determined to show a stimulatory effect on the transport of the NF-κBp65 protein from the cytoplasm to the nucleus both in vivo and in vitro, suggesting that Saa1/SAA1 could activate the NF-κB pathway.

**Conclusion:**

Taken together, our key findings highlight a novel mechanism by which silencing of SAA1 hinders PA or HFD-induced insulin resistance through inhibition of the NF-κB pathway.

**Electronic supplementary material:**

The online version of this article (10.1186/s10020-019-0075-4) contains supplementary material, which is available to authorized users.

## Background

Obesity is regarded as the major contributing factor in the development of insulin resistance (Li et al. 2015) and therefore has been the center of multiple researches due to its positive correlation with type 2 diabetes mellitus (Bessman et al. 1990; Kayaniyil et al. 2010). A high-fat diet may consequently lead to a lipotoxic effect that impairs the action of insulin and cognitive function as well as the regulatory mechanisms tasked with maintaining body weight (Cani et al. 2007; Stranahan et al. 2008). In addition, insulin resistance can be induced by palmitate (PA) in H4IIEC3 hepatocytes by reactive oxygen species, which are produced by mitochondria, a byproduct of metabolism (Nakamura et al. 2009). Although weight loss, consumption of a healthy diet and regular exercise and other lifestyle modifications can prevent the development of insulin resistance among obese individuals, these methods often fail to achieve desirable outcomes (Boden 2008). Based on a previous study, there was a persistent and profound elevation in serum amyloid A (SAA) expression in chronic inflammatory diseases, such as metabolic syndrome, obesity as well as diabetes mellitus (Wilson et al., 2018). Furthermore, SAA is found in apoB-containing lipoproteins in patients diagnosed with diabetes (Jahangiri et al. 2013). Thus, SAA could potentially be used to as a novel approach in alleviating insulin resistance in connection with SAA members with the objective of enhancing the treatment of patients with diabetes and improving their life of quality.

SAA, which is predominantly found in the bloodstream at 0.1 μM, is an acute-phase protein with apolipoprotein properties that has been implicated in the development of a number of pathological conditions, including type 2 diabetes mellitus (Baranova et al. 2005; Lakota et al. 2018). SAA, a classical, acute-phase protein and a product of hepatocytes, could potentially respond to infection, injury and inflammation and also has the ability to regulate toll-like receptor 4 (TIR4) which is associated with obesity-induced insulin resistance (Odhah et al. 2018; Sandri et al. 2008; Shi et al. 2006; Tannock et al. 2018). SAA1 has been identified in literature as a 12- to 14-kDa apolipoprotein and an acute phase reactant (Baranova et al. 2005; Hinrichs et al. 2018). A previous report has demonstrated that there exists a correlation between SAA1 genetic polymorphism and obesity among Chinese children (Zhang et al. 2013). An interesting finding suggested that there was no evidence proving that adipose tissue-derived human SAA1 could affect insulin sensitivity or obesity-associated inflammation in a mouse model (Ahlin et al. 2013). Hence, we performed microarray/based gene expression analysis, and according to the results obtained, SAA1 was identified as an obesity-related gene on the basis of the microarray data obtained from GSE39549, which was consistent with our initial hypothesis. Therefore, we came to the conclusion that SAA1 could be involved in obesity-induced insulin resistance. NF-κB, located in the downstream of interleukin-1 receptor-associated kinase 4, is comprised of a group of transcription factors (O'Reilly et al. 2014). The role of the NF-κB pathway is of great importance in the pathological development of insulin resistance in obesity and type 2 diabetes mellitus with the involvement of IKK-β (Luo et al. 2015). Another study demonstrated that SAA could influence the NF-κB pathway (Li et al. 2013). The aforementioned literature and their findings led to us drawing the hypothesis that SAA1 could be implicated in the alteration of insulin resistance with further possible involvement in the NF-κB pathway. Hence, we established PA- and high-fat diet (HFD)-induced insulin resistance cell and mouse models in order to investigate the effects of SAA1/Saa1 and the NF-κB pathway on insulin resistance.

## Methods

### Ethics statement

All experimental procedures and animal were conducted with the approval of the Animal Ethics Committee of the Second Hospital of Anhui Medical University. The present study was performed in strict accordance with the Guide for the Care and Use of Laboratory Animals of the National Institutes of Health.

#### In silico analysis

The obesity-related expression profile of GSE39549 was selected from the Gene Expression Omnibus (GEO) database (https://www.ncbi.nlm.nih.gov/geo/). The adipose tissue gene expression profile of 4 obese C57BL/6J mice induced through 24 weeks of HFD in addition to 4 C57BL/6J mice placed on a normal diet over 24 weeks were included in the differential analysis in order to screen the differentially expressed genes (DEGs) associated with obesity. The C57BL/6J mice were a branch of C57BL/6 mice (Jackson Laboratory, Bar Harbor, ME, USA). Total RNA was extracted using Trizol reagent and purified in accordance with the instructions provided on the QIAGEN RNeasy mini kit and treated with DNase I. Quality control was conducted using an Agilent Bioanalyser. The HFD-induced obesity-related DEGs were analyzed and screened out using the limma package of the R language following the chip expression matrix and the gene annotation file was downloaded. The screening criteria of DEGs was considered to be |Log fold change (FC) | > 2 and *p* < 0.05, after which the pheatmap package (https://cran.r-project.org/web/packages/pheatmap/index.htmL) was used to plot the heatmap of DEG expression. Our exploration of MEDLINE abstracts led to the finding of Digsee (http://210.107.182.61/geneSearch/), a disease gene search engine (Kim et al. 2013), which allowed us to retrieve the obesity-related genes. The interaction of DEGs and obesity-related genes was analyzed using the String database (https://string-db.org/), followed by the construction of an interaction net. The network was visualized using the Cytoscape 3.6.0 software (Shannon et al. 2003).

### Cell treatment

The human hepatoma cell line Huh7 purchased from Institute of Biochemistry and Cell Biology, Shanghai Institutes for Biological Sciences, Chinese Academy of Sciences (Shanghai, China) were cultured in Dulbecco’s modified Eagle’s medium (DMEM, Invitrogen Inc., Carlsbad, CA, USA) containing 10% fetal bovine serum (FBS), 100 units/mL penicillin and 0.1 mg/mL streptomycin. The cells were incubated at 37 °C in a humidified atmosphere containing 5% CO_2,_ with the medium changed at regular intervals of every 2 d. The Huh7 cells at the logarithmic growth phase were selected and seeded into 6-well plates at the density of 5 × 10^4^ cells/well. When cells reached 80–90% confluence, cell transfection was conducted in accordance with the instructions of the Lipofectamine 2000 (11668–027, Invitrogen Inc., Carlsbad, CA, USA). The cells were then treated with PA or BAY 11–7082 after 24 h of transfection. The complex solution composed of PA (Sigma-Aldrich Chemical Company, St Louis, MO, USA) and bovine serum albumin (BSA) was prepared according to the instructions provided by a previous literature (Cousin et al. 2001). The final concentration of PA during the cell treatment was 0.25 mM. The BAY 11–7082 was prepared at the concentration of 50 μM based on evidence from a previous literature (Lappas et al. 2005). The Huh7 cells were subsequently assigned into to blank (cells without any treatment), PA (cells with PA treatment), PA + sh-negative control (NC) (cells with PA treatment after sh-NC transfection), PA + sh-SAA1 (cells with PA treatment after sh-SAA1 transfection), PA + pcDNA3 (cells with PA treatment after pcDNA3 vectors transfection), PA + pcDNA3-SAA1 (cells with pcDNA3-SAA1 vectors transfection followed by PA treatment), PA + BAY (cells with simultaneous PA and BAY 11–7082 treatment), PA + sh-SAA1 + BAY (cells with simultaneous PA and BAY 11–7082 treatment after sh-SAA1 transfection) and PA + pcDNA3-SAA1 + BAY (cells with PA and BAY 11–7082 treatment after pcDNA3-SAA1 vector transfection) groups. All the vectors used in the present study were purchased from Shanghai GenePharma Co., Ltd. (Shanghai, China). Following treatment with PA or BAY 11–7082 for 24 h, the cells from each group were collected for subsequent experiments.

### Scintillation counting

The [^3^H]2-deoxyglucose (2DG) uptake of the cells from each group was examined based on parameters highlighted in a previous literature (Tremblay and Marette 2001). The cells that underwent varying transfections or PA treatment from each group were supplemented with 100 nM insulin, after which incubation was carried out at 37 °C for 20 min. The cells were then allocated into insulin (cells only with insulin treatment), insulin + PA, insulin + PA + sh-NC, insulin + PA + sh-SAA1, insulin + PA + pcDNA3, and insulin + PA + pcDNA3-SAA1 groups. The cells were then rinsed twice with N-2-hydroxyethylpiperazine-N′-2-ethanesulfonic acid (HEPES) buffer comprised of 20 mM HEPES (pH = 7.4), 140 mM NaCl, 5 mM KCl, 2.5 mM MgSO_4_, and 1 mM CaCl_2_, and were further cultured in HEPES buffer consisting of 10 μM [^3^H]2DG (0.5 μCi/mL) for 5 min. Finally, the cells were rinsed three times with pre-cold 0.9% NaCl and collected into 1.25 mL of 0.05 N NaOH. Cell-associated radioactivity was determined by means of scintillation counting. Protein concentration was determined using a kit (Bio-Rad Laboratories, Hercules, CA, USA) in accordance with the Bradford method. The results were expressed as pmol of the 2DG transporter per minute per milligram of cell protein (pmol of 2-DG/min/mg cell protein).

### Oil red O staining

After the attachment, the Huh7 cells were washed three times with cold phosphate buffered saline (PBS). After being fixed with 10% formalin for 10 min, the cells received a wash with PBS, and 70% ethanol. The cell monolayers were stained with oil red O solution (Sigma-Aldrich, St. Louis, MO, USA) for 15 min, followed by washing with 70% ethanol for the removal of excess stains. Finally, the cells were rinsed and differentiated with 60% isopropanol, washed with water, mounted with glycerol gelatin, and observed under an ordinary optical microscope (OLYMPUS, Tokyo, Japan). The experiment was repeated three times.

### Animal experiments

One hundred and five specific-pathogen-free (SPF) C57BL/6 mice (aged 5–6 weeks and weighing 18–22 g) were purchased from Beijing Vital River Laboratory Animal Technology Co., Ltd. (Beijing, China). Fifteen C57BL/6 mice were randomly selected and placed on a normal diet (ND group) (10% saturated fat, Research Diets: D12492), while HFD (60% saturated fat, Research Diets: D12450B) was administered in the remaining mice for a total of 16 weeks. The mice from each group were fed under 21 ± 1 °C condition with a 12-h light / 12-h dark cycle (7: 00 am to 7: 00 pm). There were three mice per cage and the mice were provided with free access to water. From the 9th week onwards, the mice in the HFD group were intraperitoneally injected with adenoviral vectors (1 × 10^9^ pfu/100 μL) or BAY-11-7082 (10 mg/kg) which was performed in line with literature procedures (Malaver et al. 2009) twice a week. Adenoviral vectors expressing shRNA against Saa1 (ad-shSaa1) and adenoviral vectors expressing Saa1 (ad-Saa1) with over 80% transfection efficiency were purchased from Shanghai Genechem Co., Ltd. (Shanghai, China) and used for transfection purposes. After 8 weeks of transfection, the liver tissues of mice were obtained and the expression of GFP in vivo was observed with the use of a fluorescence microscope (Additional file 1: Figure S1). The HFD mice were subsequently sub-grouped into the HFD (mice only treated with HFD), HFD + ad-shSaa1 (HFD + intraperitoneal injection of ad-shSaa1), HFD + BAY (HFD + intraperitoneal injection of BAY-11-7082), HFD + ad-shSaa1 + BAY (HFD + simultaneous intraperitoneal injection of ad-shSaa1 and BAY-11-7082), HFD + ad-Saa1 (HFD + simultaneous intraperitoneal injection of ad-Saa1), and HFD + ad-NC (HFD + simultaneous intraperitoneal injection of adenoviral vectors) with 15 mice each group. The weight of the mice was measured twice a week. At the end of the experiment (the 16th week), the mice were placed on a fasting diet for one night and euthanized with 1% pentobarbital sodium. The tissues were collected and stored at − 80 °C for subsequent experiments.

### Fasting blood glucose (FBG) and fasting plasma insulin (FPI) determination

At the 16th week, the glucose blood samples were obtained from the tail veins of mice after overnight fasting for the glucose assay. FBG was measured with an Accu-check Advantage glucometer and plasma glucose test strips (Roche Diagnostics GmbH, Mannheim, Germany). Plasma insulin levels were determined using an insulin enzyme-linked immunoassay (ELISA) kit with mouse insulin used as the standard (Linco Research, St Charles, MO, USA) in accordance to the manufacturer’s instructions.

### ELISA

The plasma insulin levels were determined in accordance with the instructions of the insulin ELISA kit (Linco Research, St Charles, MO, USA), while the expression of plasma Saa1 was measured according to the instructions of the Saa1 ELISA Kit (41-SAAMS-E01, ALPCO, Salem, NH, USA).

### Glucose tolerance test (GTT) and insulin resistance test

At the 16th week, GTT was conducted after the mice were made to fast for one night. The mice were intraperitoneally injected with glucose at dosage of 1.5 g/kg body weight. FBG levels were examined at baseline as well as at the 30, 60, 90, and 120 min time points post administration. For the insulin resistance test, the mice were made to fast for one night prior to the intraperitoneal injection of 0.75 IU/kg insulin, while the FBG levels were assessed at baseline as well as at the 30, 60, 90, and 120 min time points post insulin injection. The insulin resistance index was calculated based on the level of FBG and FPI: homeostasis model assessment of insulin resistance (HOMA-insulin resistance) = FBG × FPI / 22.5.

### Reverse transcription quantitative polymerase chain reaction (RT-qPCR)

The frozen kidney, heart, muscle and liver tissues (20 mg) were grinded in the mortar that had been pretreated with liquid nitrogen into powder, after which it was promptly transferred to the micro-tissue homogenizer that had been pretreated with a cold ice bath. Following the addition of 1 mL Trizol reagent (Invitrogen Inc., Carlsbad, CA, USA), the samples were well homogenized until it was observed to be thoroughly cleared, after which it was placed on ice for 10 min and centrifugation was carried out at 25764×g for 10 min at 4 °C. Afterwards, total RNA was extracted from the tissues (the supernatant after homogenate and centrifugation) and cells from each group using Trizol RNA (Invitrogen Inc., Carlsbad, CA, USA). In accordance with the instructions of the kit (Fermentas Inc., Hanover, MD, USA), RNA reverse transcription was performed using the two-step method. The reaction conditions were performed as follows: at 70 °C for 10 min, ice-bathed for 2 min, at 42 °C for 60 min and at 70 °C for 10 min. The cDNA that was obtained was temporarily stored at − 80 °C. The RT-qPCR reaction system was conducted according to instructions of the kit (Fermentas Inc., Hanover, MD, USA). The primer sequences employed are depicted in Table 1. The reaction conditions consisted of pre-denaturation at 95 °C for 30 s, followed by 40 cycles of denaturation at 95 °C for 10 s, annealing at 60 °C for 20 s and extension at 70 °C for 10 s. Finally, the mRNA expression of each gene was determined using a real-time fluorescence qPCR instrument (Bio-Rad iQ5, Bio-Rad Laboratories, Hercules, CA, USA). β-actin was considered to be the internal reference. The relative expression of gene was calculated using the 2^-ΔΔCt^ method. The experiment was repeated three times in an independent manner.

**Table 1:** Primer sequence for RT-qPCR

Gene	Forward (5′-3′)	Reverse (5′-3′)
SAA1 (human)	CCAATCTCCGACCTGCTG	GCTTTGTATCCCTGCCCTGAG
Saa1 (mouse)	GGACTGCCTGACAAATACTGAG	GAGCATCTTCAGTGTTCCTAGG
IRS1 (human)	AGGATATTTAATTTGCCTCGG	AAGCGTTTGTGCATGCTCTTG
SOCS3 (human)	CCACTCTTCAGCATCTCTGT	ACTGAACCTGACCGTACAATCGTACTGGTCCAGGAACT
β-actin (human)	GCAAAGACCTGTACGCCAACA	TGCATCCTGTCGGCAATG
β-actin (mouse)	GACATGGAGAAGATCTGG	CTTTACGGATGACAACG

Notes: *RT-qPCR* reverse transcription quantitative polymerase chain reaction, *Saa1* serum amyloid A protein 1, *IRS1* insulin receptor substrate 1, *SOCS3* suppressor of cytokine signaling 3

### Western blot analysis

The kidney, heart, muscle, and liver tissues (10 mg) of mice received three washes with saline containing protease inhibitor phenylmethanesulfonyl fluoride (PMSF, final concentration 1 mM), and transferred into a 1.5 mL Eppendorf (EP) tube, followed by the addition of 500 μL of mixture of Radio-Immunoprecipitation assay (RIPA) protein lysis buffer and PMSF at the ratio of 100: 1. Afterwards, the tissues were fully shredded with sterile anatomical scissors, homogenized in a precooled glass homogenizer pretreated with an ice bath and lysed in the ice water mixture for 30 min. During this process, the micrometer was employed to triturate several times in an intermittent manner. The homogenate of tissues in each group was transferred to fresh 1.5 mL EP tubes and centrifuged at 25764×g for 40 min at 4 °C. Cell extracts of the nucleus and cytoplasm were obtained on the basis of a procedure demonstrated in a previous literature (Taddeo et al. 2003). The protein concentration of each sample was determined using a bicinchoninic acid (BCA) kit (Sigma-Aldrich Chemical Company, St Louis, MO, USA). Furthermore, 20 μg of protein was blotted onto the gel, separated with 10% sodium dodecyl sulfate-polyacrylamide gel electrophoresis (SDS-PAGE), and was transferred onto a polyvinylidene fluoride (PVDF) membrane (Millipore Corp., Bedford, MA, USA). Next, membrane blockade was performed using 5% skimmed milk at room temperature for 1 h, and then washed once with PBS. Subsequently, the cells were incubated overnight at 4 °C following the addition of the diluted primary antibody, rabbit polyclonal antibodies to SAA1 (ab171030, 1: 1000), β-actin (ab8227, 1: 1000), IRS1 (ab131487, 1: 1000), SOCS3 (ab16030, 1: 1000), p65 (ab16502, 1: 500), laminA/C (ab227176, 1: 500) and phosphorylated IRSI (p-IRS1; phospho Y896; ab4873, 1: 1000). The membrane was then washed three times with PBS (5 min each) at room temperature, and was added with the secondary antibody, horseradish peroxidase (HRP)-labeled goat anti-rabbit antibody to IgG (ab97051, 1: 200) for incubation at 37 °C for 1 h, followed by three washes with PBS at room temperature (5 min each). All antibodies were purchased from Abcam Inc. (Cambridge, MA, USA) with the exception of p-IRS1 (Cell Signaling Technologies, Beverly, MA, USA). The membrane was soaked in enhanced chemiluminescent (ECL) solution (Pierce, Rockford, IL, USA) at room temperature for 1 min following liquid aspiration. Next, the membrane was covered using a plastic wrap. Finally, the membrane was exposed under dark conditions, developed, and photographed, after which the results were analyzed accordingly. Finally, the gray values of the target bands were analyzed using Image J software, with the experiment repeated three times in an independent fashion.

### Statistical analysis

SPSS 21.0 statistical software (IBM Corp. Armonk, N.Y., USA) was employed for statistical analysis. The measurement data were expressed as mean ± standard deviation. Differences between two groups were conducted using the *t* test. Comparisons among multiple groups were performed with one-way analysis of variance (ANOVA) while the Welch method was applied in the event of an uneven variance. Multiple comparisons between groups were analyzed using *Dunnetts’T3* test. A value of *p* < 0.05 was considered to be statistically significant.

## Results

### Screening of obesity-related DEGs through in silico analysis

**Fig. 1:**
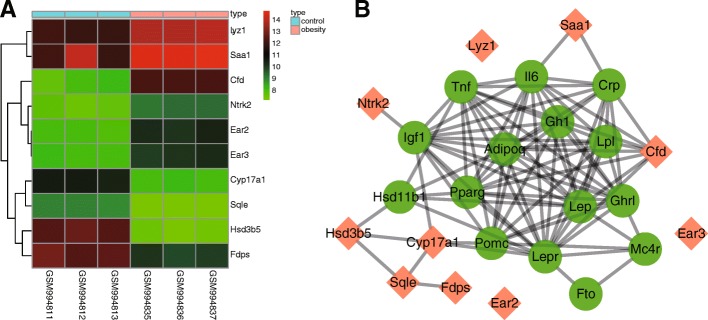
SAA1 is associated with HFD-induced obesity. **a**, the heatmap of the top 10 DEGs screened out from the GSE39549 dataset. The abscissa indicates the sample number, the ordinate indicates the DEGs, and the upper right histogram is the color gradation. Each rectangle corresponds to value of one sample expression; **b**, the interaction network between the DEGs and obesity-related genes. Orange indicates DEGs and green indicates obesity-related genes. Cyp17a1, cytochrome P450, family 17, subfamily a, polypeptide 1; Cfd, complement factor D; SAA1, serum amyloid A protein 1; DEGs, differentially expressed genes

Initially, DEGs were screened out from the GSE39549 chip, based on the criteria *p* < 0.05 and |LogFC| > 2 with the heatmap of the top 10 DEGs (Hsd3b5, Cyp17a1, Cfd, Sqle, Ear2, Fdps, Ear3, Lyz1, Ntrk2, and Saa1) plotted (Fig. 1a). Obesity-related genes were retrieved in the Digsee database and the top 15 obesity-related genes were selected as disease genes (Adipoq, Igf1, Fto, Hsd11b1, Tnf, Gh1, Mc4r, Lep, Pparg, Il6, Crp, Lepr, Pomc, Lpl, and Ghr1). The interaction between top 10 DEGs and obesity-related genes was analyzed using the String database, and the interaction network was constructed (Fig. 1b). The DEGs Cyp17a1, Cfd and Saa1 were the ones that interacted with obesity-related genes in the interaction network, on the basis of which we speculated that these genes may be related to obesity. The information from the gene function was retrieved from the National Center for Biotechnology Information Search database (NCBI; https://www.ncbi.nlm.nih.gov/), which revealed that complement factor D (Cfd) was a cell signaling protein secreted by adipocytes, which could potentially regulate insulin secretion in a mouse. Cytochrome P450, family 17, subfamily A, polypeptide 1 (Cyp17A1), a member of the CYP450 family, is implicated in cholesterol, steroids and other lipid responses, and their relationship with obesity has been previously reported (Mathews et al. 2014; Rosen et al. 1989; Tabur et al. 2016; Yousefi et al. 2011). Saa1, a member of the serum amyloid family, was also found to exhibit higher expression in obese mice compared with the control group in the heatmap (Fig. 1a). Furthermore, a previous study indicated that inhibition of the NF-κB pathway could relieve HFD-induced obesity and poor glucose intolerance (Benzler et al. 2015), and that Saa1 could activate the NF-κB pathway (Siegmund et al. 2016). Based on these findings, we drew the hypothesis that Saa1 could mediate the NF-κB pathway in HFD-induced obesity.

### SAA1 silencing suppresses PA-induced insulin resistance in Huh7 cells

**Fig. 2:**
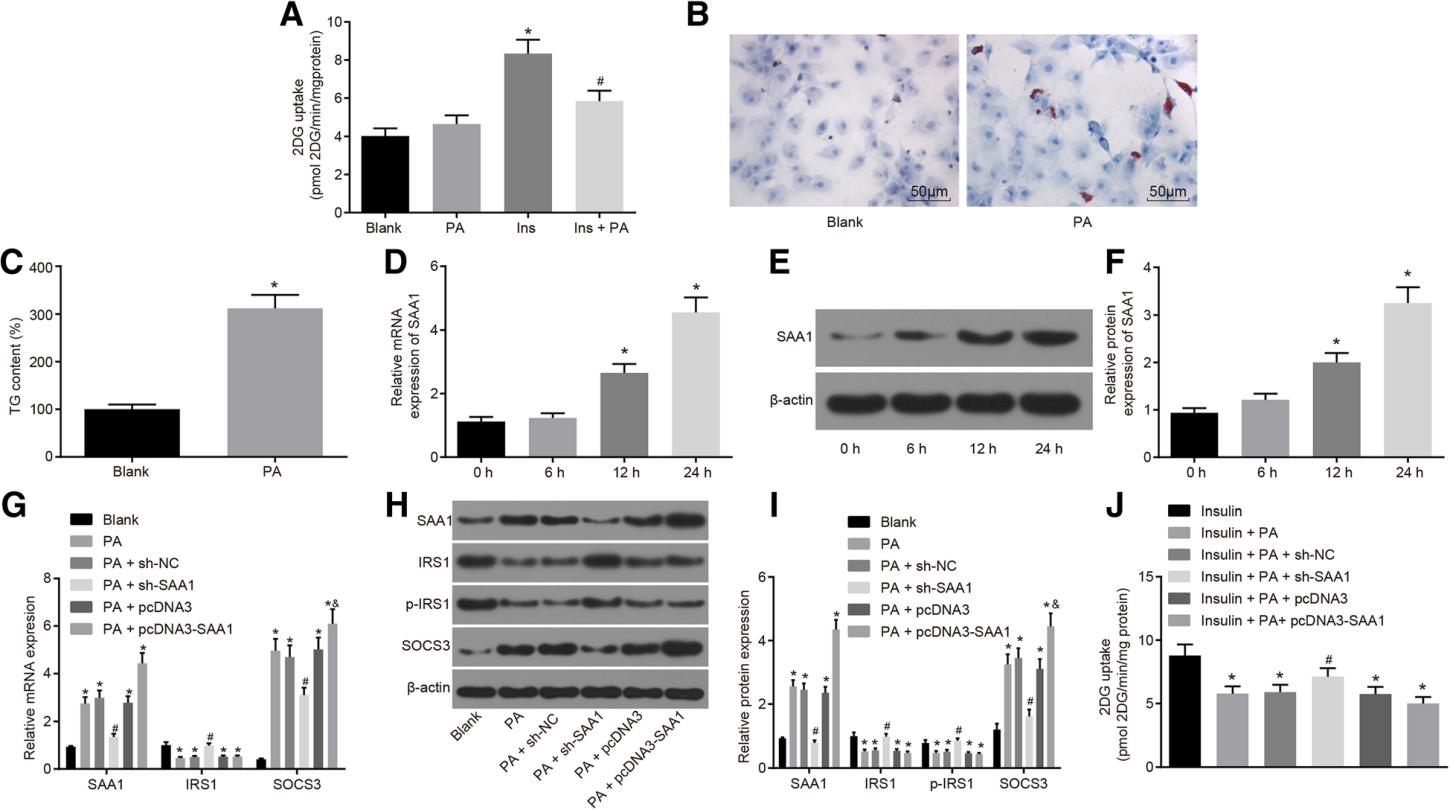
The insulin resistance in Huh7 cells is suppressed by silencing of SAA1. **a**, insulin-stimulated 2DG uptake in response to the treatment of PA, insulin and insulin + PA; *, *p* < 0.05 vs. the blank group; #, *p* < 0.05 vs. the insulin group; **b**, Huh7 cell images after PA treatment by oil red O staining (× 200); **c**, the contents of triglycerides after PA treatment; **d**, the mRNA expression of SAA1 in PA-treated Huh7 cells according to RT-qPCR; *, *p* < 0.05 vs. the 0-h group; **e**, the gray value of SAA1 and β-actin protein bands measured by western blot analysis; **f**, the protein expression of SAA1 in PA-treated Huh7 cells according to western blot analysis; *, *p* < 0.05 vs. the 0-h group; **g**, the mRNA expression of SAA1, IRS1 and SOCS3 in response to the treatment of PA + sh-SAA1, PA + pcDNA3, PA + pcDNA3-SAA1 tested by RT-qPCR; *, *p* < 0.05 vs. the blank group; #, *p* < 0.05 vs. the PA + sh-NC group; &, *p* < 0.05 vs. the PA + pcDNA3 group; **h**, the gray value of SAA1, IRS1, SOCS3, and p-IRS1 protein bands evaluated by western blot analysis; **i**, the protein expression of SAA1, IRS1 and SOCS3, and the extent of IRS1 phosphorylation in response to the treatment of PA + sh-SAA1, PA + pcDNA3, PA + pcDNA3-SAA1 according to western blot analysis; *, *p* < 0.05 vs. the blank group; #, *p* < 0.05 vs. the PA + sh-NC group; &, *p* < 0.05 vs. the PA + pcDNA3 group; **j**, insulin-stimulated 2DG uptake in response to the treatment of insulin + PA + sh-SAA1, insulin + PA + pcDNA3, insulin + PA + pcDNA3-SAA1; *, *p* < 0.05 vs. the insulin group; #, *p* < 0.05 vs. the insulin + PA + sh-NC group; data were expressed by means ± standard deviation; differences between two groups were compared by the *t* test; multiple groups were compared by one-way analysis of variance; the experiment was repeated 3 times; SAA1, serum amyloid A protein 1; 2DG, [^3^H]2-deoxyglucose uptake; PA, palmitate; RT-qPCR, reverse transcription quantitative polymerase chain reaction; SOCS3, suppressor of cytokine signaling 3; IRS1, insulin receptor substrate 1; p-IRS1, phosphorylation level of IRS1; NC, negative control

Obesity is often associated with insulin resistance and abnormal glucose homeostasis (Hotamisligil et al. 1995). The main association between obesity and type 2 diabetes mellitus lies on insulin resistance that is believed to be initiated by obesity (Pandolfi et al. 1994). In the present study, we investigated the role of SAA1 in insulin resistance by establishing PA-induced cell insulin resistance models. When the cell model was identified, the results showed no significant difference in 2DG uptake between the PA group and the blank group, while the 2DG uptake in the insulin group was significantly higher by approximately 2 folds (*p* < 0.05). In contrast to the insulin group, there was a significant decrease of up to 30% in 2DG uptake in the insulin + PA group (Fig. 2a). The results of oil red O staining revealed that the number of bright red lipid droplets was significantly increased in the PA group, while this number was far less in the normal cells (Fig. 2b). In comparison with the blank group, triglycerides (TG) in Huh7 cells that had been treated with PA increased by nearly three folds (*p* < 0.05; Fig. 2c). These results were highly indicative of the successful establishment of the PA-induced cell insulin resistance models.

In order to further investigate the effect of SAA1 on PA-induced insulin resistance, SAA1 was overexpressed or silenced in Huh7 cells. The results revealed that the expression of SAA1 in Huh7 cells was gradually promoted by PA treatment as the culture time prolonged. When compared with cells treated with PA for 0 h, the expression of SAA1 was significantly increased at the time period between 12 h - 24 h following PA treatment (*p* < 0.05) (Fig. 2d, e and f). Determination of insulin resistance-related genes revealed that compared with the blank group, PA treatment significantly enhanced the expression of SAA1 and SOCS3, suppressed the expression of IRS1 and also, exhibited a reduction in the extent of IRS1 phosphorylation (all *p* < 0.05) (Fig. 2g, h and i). Compared with the PA + sh-NC group, there was higher expression of IRS1 and lower SAA1 and SOCS3 expression in the PA + sh-SAA1 group, and there was an evident increase in the extent of IRS1 phosphorylation (all *p* < 0.05) (Fig. 2g, h and i). There was a remarkable increase in the expression of SAA1 and SOCS3 in the PA + pcDNA3-SAA1 group compared with the PA + pcDNA3 group (both *p* < 0.05) (Fig. 2g-i). Measurement of the sensitivity of SAA1 to insulin revealed that 2DG uptake was markedly reduced in the insulin + PA group and the insulin + PA + pcDNA3-SAA1 group compared with the insulin group, while the 2DG uptake was markedly elevated in the insulin + PA + pcDNA3-SAA1 group compared with the insulin + PA + sh-NC group (*p* < 0.05) (Fig. 2j). These findings suggest that SAA1 gene silencing can result in the suppression of insulin resistance in PA-induced insulin resistance model in Huh7 cells.

### Silencing of SAA1 suppresses PA-induced insulin resistance through the NF-κB pathway in Huh7 cells

**Fig. 3:**
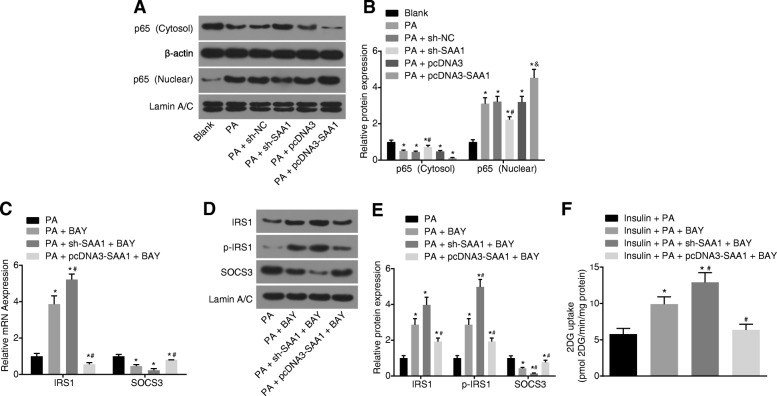
Silencing of SAA1 exerts an inhibitory effect over PA-induced insulin resistance via the activation of the NF-κB pathway. **a**, the gray value of cytoplasmic p65 and nucleus p65 protein bands according to the western blot analysis; **b**, the protein expression of cytoplasmic p65 and nucleus p65 in response to the treatment of PA + sh-SAA1, PA + pcDNA3, PA + pcDNA3-SAA1; *, *p* < 0.05 vs. the blank group; #, *p* < 0.05 vs. the PA + sh-NC group; &, *p* < 0.05 vs. the PA + pcDNA3 group; **c**, the mRNA expression of IRS1 and SOCS3 in response to the treatment of PA + BAY, PA + sh-SAA1 + BAY, PA + pcDNA3-SAA1 + BAY determined by RT-qPCR; *, *p* < 0.05 vs. the PA group; #, *p* < 0.05 vs. the PA + BAY group; **d**, the gray value of IRS1, p-IRS1 and SOCS3 protein bands in response to the treatment of PA + BAY, PA + sh-SAA1 + BAY, PA + pcDNA3-SAA1 + BAY according to the western blot analysis; **e**, the protein expression of IRS1 and SOCS3, and the extent of IRS1 phosphorylation in response to the treatment of PA + BAY, PA + sh-SAA1 + BAY, PA + pcDNA3-SAA1 + BAY according to western blot analysis; *, *p* < 0.05 vs. the PA group; #, *p* < 0.05 vs. the PA + BAY group; **f**, 2DG uptake in response to the treatment of insulin + PA + BAY, insulin + PA + sh-SAA1 + BAY, insulin + PA + pcDNA3-SAA1 + BAY; *p* < 0.05 vs. the insulin + PA group; #, *p* < 0.05 vs. the insulin + PA + BAY group; data were expressed by means ± standard deviation; multiple groups were compared by one-way analysis of variance; the experiment was repeated 3 times; SAA1, serum amyloid A protein 1; NF-κB, nuclear factor-κB; 2DG, [3H]2-Deoxyglucose Uptake; PA, palmitate; RT-qPCR, reverse transcription quantitative polymerase chain reaction; SOCS3, suppressor of cytokine signaling 3; IRS1, insulin receptor substrate 1; p-IRS1, phosphorylated IRS1; NC, negative control

PA-induced cell insulin resistance models were established in order to ascertain as to whether SAA1 was associated with the NF-κB pathway in PA-induced insulin resistance. The results obtained indicated that compared with the blank group, there was a significant decrease in the cytoplasmic p65 protein in the PA group, while the p65 protein in the nucleus increased (both *p* < 0.05) (Fig. 3a and b). Compared with the PA + sh-NC group, the PA + sh-SAA1 group displayed significantly elevated cytoplasmic p65 protein and down-regulated p65 protein in the nucleus, while the PA + pcDNA3-SAA1 group illustrated significantly increased p65 protein in the nucleus versus the PA + pcDNA3 group (all *p* < 0.05) (Fig. 3a and b). These results demonstrated that there could potentially be a correlation between SAA1 and the NF-κB pathway in Huh7 cells.

When the NF-κB pathway was inhibited as a result of treatment with BAY 11–7082, the results revealed that compared with the PA group, the expression of IRS1 and the extent of IRS1 phosphorylation significantly increased, while SOCS3 markedly decreased in the PA + BAY group (all *p* < 0.05). Compared with the PA + BAY group, higher expression of IRS1 and lower SOCS3 exhibited in the PA + sh-SAA1 + BAY group, and there was a significant increase in the extent of IRS1 phosphorylation (all *p* < 0.05). An opposite trend was found in the PA + pcDNA3-SAA1 + BAY group (Fig. 3c, d and e). The 2DG uptake was remarkably upregulated in the insulin + PA + BAY group relative to the insulin + PA group (*p* < 0.05). The insulin + PA + sh-SAA1 + BAY group exhibited a significant increase in 2DG uptake in contrast to the insulin + PA + BAY group, while 2DG uptake decreased in the insulin + PA + pcDNA3-SAA1 + BAY group (both *p* < 0.05) (Fig. 3f). These findings demonstrated that silencing of SAA1 resulted in the inhibition of insulin resistance in PA-treated Huh7 cells through the inhibition of the NF-κB pathway.

### Silencing of Saa1 inhibits glucose tolerance and systemic insulin sensitivity in HFD-induced obesity through the NF-κB pathway

**Fig. 4:**
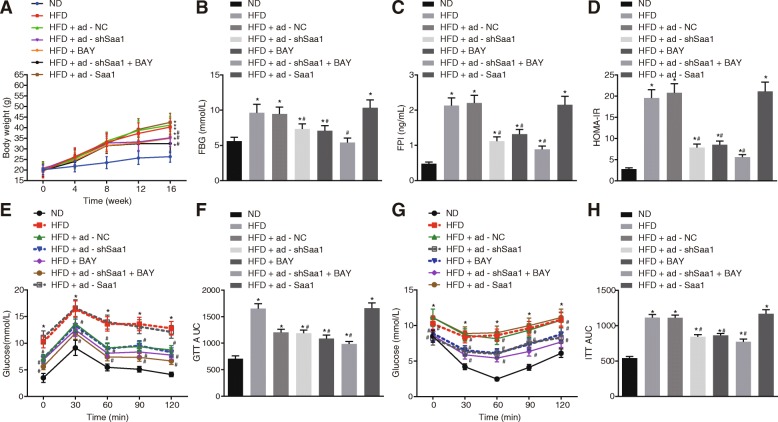
Glucose tolerance and insulin sensitivity in HFD mice are inhibited by silencing of Saa1. **a**, body weight changes of HFD mice in response to the treatment of HFD + ad-shSaa1, HFD + BAY, HFD + ad-shSaa1 + BAY and HFD + ad-Saa1 at the 0th, 4th, 8th, 12th and 16th week; **b**, the FBG of HFD mice after 12-h fasting in response to the treatment of HFD + ad-shSaa1, HFD + BAY, HFD + ad-shSaa1 + BAY and HFD + ad-Saa1; **c**, the FPI of HFD mice after 12-h fasting in response to the treatment of HFD + ad-shSaa1, HFD + BAY, HFD + ad-shSaa1 + BAY and HFD + ad-Saa1; **d**, HOMA-insulin resistance of HFD mice in response to the treatment of HFD + ad-shSaa1, HFD + BAY, HFD + ad-shSAA1 + BAY and HFD + ad-SAA1; **e**, the blood glucose levels of HFD mice in response to the treatment of HFD + ad-shSaa1, HFD + BAY, HFD + ad-shSaa1 + BAY and HFD + ad-Saa1 according to GTT; **f**, AUC of HFD mice in response to the treatment of HFD + ad-shSaa1, HFD + BAY, HFD + ad-shSaa1 + BAY and HFD + ad-SAA1; **g**, blood glucose levels of HFD mice in response to the treatment of HFD + ad-shSaa1, HFD + BAY, HFD + ad-shSaa1 + BAY and HFD + ad-Saa1 corresponding to insulin resistance test; **h**, AUC of HFD mice in response to the treatment of HFD + ad-shSaa1, HFD + BAY, HFD + ad-shSaa1 + BAY and HFD + ad-Saa1 corresponding to insulin resistance test; data were expressed by means ± standard deviation; multiple groups were compared by one-way analysis of variance; *n* = 15; *, *p* < 0.05 vs. the ND group; #, *p* < 0.05 vs. the HFD group. Saa1, serum amyloid A protein 1; ND, normal diet; HFD, high-fat diet; GTT, glucose tolerance test; AUC, area under the curve; NC, negative control; FPI, fasting plasma insulin; FBG, fasting blood glucose; HOMA-insulin resistance, homeostasis model assessment of insulin resistance

HFD-induced mice were employed to establish insulin resistant mouse models in order to elucidate the role of Saa1 in insulin resistance in vivo. The measurement of body weight, FBG, FPI, and HOMA-insulin resistance was conducted, and the results revealed that there was a significant enhancement in the body weight, FBG, FPI, and HOMA-insulin resistance of mice in the HFD-induced mice than in the ND group by 1.53, 1.72, 4.44 and 7.04 times respectively on the 16th week (all *p* < 0.05) (Fig. 4a ,b, c and d). These findings indicated the successful establishment of the HFD-induced insulin resistance mice models.

In addition, the expression of Saa1 among the mouse models was determined. The results revealed that the expression of Saa1 in kidney, heart, muscle, and liver tissues of the HFD group was significantly higher than that in the ND group (all *p* < 0.05) (Additional file 2: Figure S2A). The expression of Saa1 in plasma in the HFD group was significantly higher than that in the ND group (*p* < 0.05) (Additional file 2: Figure S2D). Saa1 was overexpressed or silenced in mice through tail venous injection of adenoviral vectors. The results demonstrated that compared with the ad-NC group, the expression of Saa1 significantly increased in the liver of mice in the ad-Saa1 group, while it decreased in the ad-shSaa1 group (both *p* < 0.05) (Additional file 3: Figure S3). The body weight, FBG, FPI and HOMA-insulin resistance of mice in the HFD + ad-shSaa1, HFD + BAY and HFD + ad-shSaa1 + BAY groups significantly decreased in contrast to the HFD group (all *p* < 0.05). There were no significant differences detected in terms of body weight, FBG, FPI and HOMA-insulin resistance between the HFD + ad-Saa1 group and the HFD group (all *p* > 0.05) (Fig. 4a, b, c and d). In addition, we measured insulin sensitivity in mice by GTT and insulin resistance test. Following the injection of glucose or insulin, the mice were found to have a significant increase in FBG and the area under the curve (AUC) in the HFD group than those in the ND group (*p* < 0.05) (Fig. 4e and f). FBG and AUC in the HFD + ad-shSaa1 group, HFD + BAY, and HFD + ad-shSaa1 + BAY groups were significantly lower than those in the HFD group (all *p* < 0.05) (Fig. 4g and h). The aforementioned findings suggested that the silencing of Saa1 or inhibition of the NF-κB pathway could result in the inhibition of insulin resistance in HFD-induced mice.

### Saa1 activates the NF-κB pathway in HFD-induced obesity

**Fig. 5:**
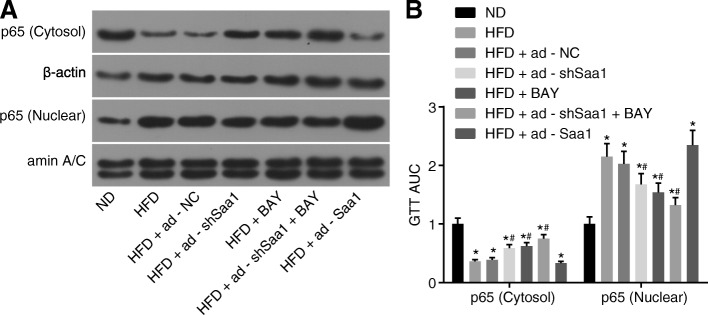
NF-κB pathway is activated by Saa1 in HFD mouse models. **a**, the grey value of cytoplasmic p65 and nucleus p65 protein bands in liver tissues of HFD mice after fed for 16 weeks, determined by western blot analysis; **b**, the protein expression of cytoplasmic p65 and nucleus p65 in liver tissues of HFD mice; *, *p* < 0.05 vs. the ND group; #, *p* < 0.05 vs. the HFD group; data were expressed by means ± standard deviation; multiple groups were compared by one-way analysis of variance; n = 15; NF-κB, nuclear factor-κB; SAA1, serum amyloid A protein 1; ND, normal diet; HFD, high-fat diet; NC, negative control

Finally, the effect of Saa1 on the NF-κB pathway in liver tissues of mice in each group was examined in order to determine whether Saa1 could regulate the NF-κB pathway. The results obtained suggested that compared with the ND group, there was a remarkable reduction in the cytoplasmic p65 protein in the HFD group, while the expression of p65 protein in the nucleus significantly increase (all *p* < 0.05)d. Compared with the HFD group, cytoplasmic p65 protein in the HFD + ad-shSaa1, HFD + BAY, and HFD + ad-shSaa1 + BAY groups was significantly increased, while the expression of the p65 protein in the nucleus was significantly decreased (all *p* < 0.05) (Fig. 5). These results indicated that Saa1 exerted its effect by activating the NF-κB pathway in mice liver.

## Discussion

Insulin resistance shares a strong association with type 2 diabetes mellitus (Perry et al. 2014). The liver plays a crucial contributory role in the event of insulin degradation, as it mediated the delivery of insulin to extrahepatic organs (Jin et al. 2015), along with its function as a key regulatory organ system in relation to metabolism as well as the action of insulin (Miao et al. 2014). A previous study used PA to treat Huh7 human hepatoma cells for the purpose of establishing an insulin resistant cell model (Arturi et al. 2011). Huh7 cells were used to establish insulin resistant cell model in the present study. Our study was conducted with a primary objective of exploring the direct effects of SAA1 silencing on insulin resistance, which is known to be induced by obesity. The key findings from this study revealed that the inactivation of the NF-κB pathway and silencing of SAA1 exert a positive effect on insulin resistance through the enhancement of insulin sensitivity both in vitro and in vivo.

Zhang et al. conducted a survey of 520 Chinese children and the results of which revealed that there is a high expression in circulating SAA in obese children, indicating that SAA1 gene polymorphism is associated with obesity in Chinese children (Zhang et al. 2013). However, there’s very little known regarding the underlying biological functions and molecular regulatory mechanisms of SAA1. The initial findings of our study demonstrated that a decrease in the expression of SAA1 could improve glucose tolerance in mice on HFD; moreover, there exists a positive correlation between SAA1 and insulin resistance and that the silencing of SAA1 results in an increase in insulin sensitivity. Higher SAA1 levels under long-term HFD treatment could lead to extensive SAA1-derived amyloid deposits, which may consequently lead to the complications associated with HFD-induced obesity and metabolic disorders (Jang et al. 2016). Based on a previous study, SAA is considered to be responsible for insulin resistance and type 2 diabetes mellitus (Xie et al. 2013). Existing literature has illustrated that SAA is positively associated with insulin sensitivity in adipocytes (Ye et al. 2009). Another study also demonstrated that there is a positive association between SAA and insulin sensitivity and that an increase SAA level could result in insulin resistance in human adipose tissues, which was consistent with our findings (Filippin-Monteiro et al. 2012). Ahlin et al. have provided strong evidence that adipose tissue-derived human SAA had no significant effect on the development of atherosclerosis in mice (Ahlin et al. 2014). However, the absence of patient in clinical response for SAA treatment means that this finding can’t be fully validated. This finding further highlights on the underlying correlation of SAA with insulin resistance. In addition, our study also revealed that there is an inverse relationship between SAA1 and the expression of IRS1 while it positively regulates the expression of SOCS3. IRS1 is a key signaling protein involved in insulin signal transduction in adipose tissue and its expression can be downregulated by SAA1 (Ye et al. 2009). As it is influenced by inflammation, the reduction in the levels of SOCS3 has been reported to be a protective factor that acts to prevent obesity-induced insulin sensitivity (Jorgensen et al. 2013; Palanivel et al. 2012). Similarly, under the mediation of IL-6, SAA was capable of facilitating SOCS3 expression while lowering IRS1 levels in muscle (Zhang et al. 2009).

Heyman-Lindén et al. demonstrated that there existed a correlation between SAA protein and the NF-κB/STAT3 pathway, and proved that the activation of the NF-κB/STAT3 pathway induced by HFD results in the up-regulation of SAA protein expression (Heyman-Linden et al. 2016), but this finding is yet to be confirmed in detail. However, during the current study, we found that SAA1 silencing resulted in the downregulation of the NF-κB pathway in insulin resistance, which was further reflected by reductions in p65 protein expression. PA can induce insulin resistance through multiple pathways, including reticulum (ER) stress, insulin signaling and inflammation (Hage Hassan et al. 2012, Mazibuko et al. 2015, Ramkhelawon et al. 2014). Previous reports have indicated that exogenous PA is different from endogenous PA in relation to the inhibition of the insulin pathway. The overexpression of SAA1 has been shown to have no effect on the extent of IRS1 phosphorylation, with studies demonstrating that SAA1 was involved in insulin resistance through other mechanisms, as opposed to that of exogenous PA (Wei et al. 2016). During the process of PA-induced insulin resistance in myotube models, the NF-κB transcription played a role as a key mediator (Ajuwon and Spurlock 2005). Meanwhile, SAA has been reported to activate the NF-κB signaling pathway in human and murine intestinal epithelial cells (Jijon et al. 2005). Jitpean S et al. demonstrated that the SAA member SAA3, in a MyD88-dependent way, was capable of activating the NF-κB pathways (Deguchi et al. 2013). In addition, SAA can activate the NF-κB signaling pathway and SAA in response to IL-1 + 6 is caused by combining nuclear translocation of NF-κB p65 and the phosphorylation of STAT3 (Hagihara et al. 2005; Jijon et al. 2005), which was also found to be consistent with our findings. Furthermore, inhibiting the NF-κB pathway can alleviate obesity and glucose intolerance induced by a diet high in fat (Benzler et al. 2015), while SAA has been proven to activate the NF-κB pathway (Siegmund et al. 2016). These findings collectively found that there is a high expression of Saa1 in obese mice, and silencing of Saa1 or the treatment of BAY 11–7082 could improve glucose tolerance and systemic insulin sensitivity of mice fed with HFD.

## Conclusions

**Fig. 6:**
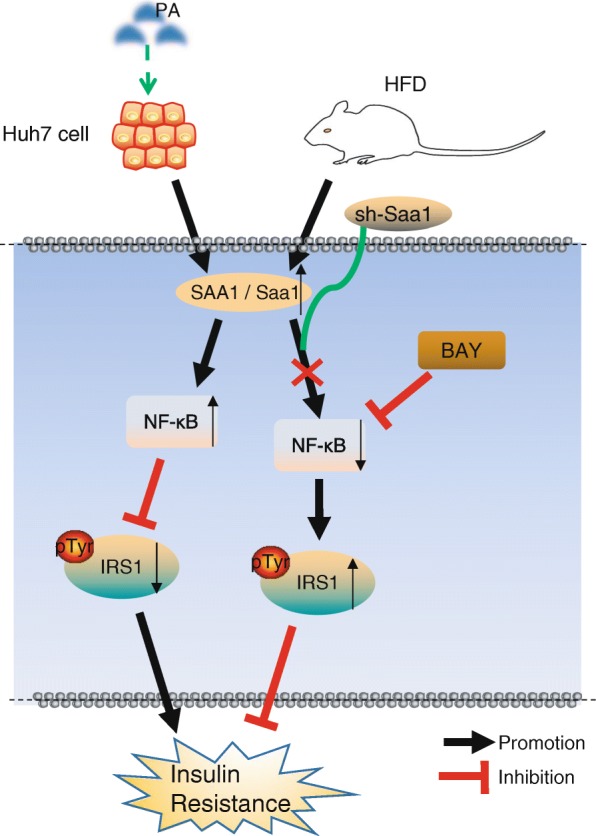
Molecular mechanism map of SAA1 involved in insulin resistance by activating the NF-κB pathway. SAA1 mediates PA- or HFD-induced insulin resistance though the activation of the NF-κB pathway. In PA-or HFD-induced insulin resistance models, up-regulation of SAA1 could inhibit the activation of IRS1 signaling by activating the NF-κB pathway, which contributes to insulin resistance. The silencing of SAA1 or treatment with BAY 11–7082 (NF-κB inhibitor) can relieve PA- or HFD-induced insulin resistance by inhibiting the NF-κB signaling pathway and IRS1 signaling. SAA1, serum amyloid A protein 1; IRS1, insulin receptor substrate 1; NF-κB, nuclear factor-κB; PA, palmitate; HFD, high-fat diet

Our study found that the expression of Saa1 in kidney, heart, muscle, liver and plasma of HFD-induced mice was significantly increased. Injection of Saa1 inhibitor via caudal vein could effectively inhibit Saa1 and insulin resistance induced by obesity, suggesting that Saa1 inhibitor may be a potentially effective therapeutic strategy for obesity-induced insulin resistance (Fig. 6). However, more large scale studies are required in terms of collecting information on insulin resistance patients and dividing them into obese and non-obese groups to explore the difference of SAA1, the pathological and clinical features between the two groups.

## Additional files

Additional file 1: **Figure S1.** After 8 weeks of transfection, the liver tissue of mice was obtained and the expression of GFP in vivo was observed under a fluorescence microscope (× 200). (EPS 6514 kb)

Additional file 2: **Figure S2.** Saa1 is highly expressed in mouse models of obesity-induced insulin resistance. A, the mRNA expression of Saa1 in kidney, heart, muscle, and liver tissues of HFD-induced mouse models according to RT-qPCR; B, the gray value of Saa1 and β-actin protein bands according to western blot analysis; C, the protein expression of Saa1 in kidney, heart, muscle, and liver of HFD-induced mouse models according to western blot analysis; D, Saa1 expression in plasma of mouse models; *, *p* < 0.05 vs. the ND group; data were expressed by means ± standard deviation; differences between two groups were compared by *t* test; *n* = 15; Saa1, serum amyloid A protein 1; RT-qPCR, reverse transcription quantitative polymerase chain reaction; ND, normal diet; HFD, high-fat diet. (EPS 3013 kb)

Additional file 3: **Figure S3.** Overexpression of Saa1 or silencing of Saa1 in mice. A, the mRNA expression of Saa1 in mouse liver tissues after venous injection of adenoviral vector into tail; B, the gray value of Saa1 and β-actin protein bands in mouse liver tissues after venous injection of adenoviral vector into tail; C, the protein expression of Saa1 in mouse liver tissues after injection of adenoviral vector via tail vein; *, *p* < 0.05 vs. the HFD + ad-NC group; data were expressed by means ± standard deviation; multiple groups were compared by one-way analysis of variance; *n* = 15; Saa1, serum amyloid A protein 1; ND, normal diet; HFD, high-fat diet; NC, negative control. (EPS 1733 kb)

## Abbreviations

ANOVA: Analysis of variance
BSA: Bovine serum albumin
CST: Cell Signaling Technologies
ECL: Enhanced chemiluminescent
ELISA: Enzyme-linked immunoassay
FBG: Fasting blood glucose
FBS: Fetal bovine serum
FPI: Fasting plasma insulin
GTT: Glucose tolerance test
HFD: High-fat diet
NC: Negative control
PA: Palmitate
PVDF: Polyvinylidene fluoride
SAA1: Serum amyloid A-1
SPF: Specific pathogen-free
TIR4: Toll-like receptor 4
